# Real-Time UV-Visible Spectroscopy Analysis of Purple Membrane-Polyacrylamide Film Formation Taking into Account Fano Line Shapes and Scattering

**DOI:** 10.1371/journal.pone.0110518

**Published:** 2014-10-17

**Authors:** María Gomariz, Salvador Blaya, Pablo Acebal, Luis Carretero

**Affiliations:** Departamento de Ciencia de Materiales, Óptica y Tecnología Electrónica, Universidad Miguel Hernández, Elx (Alicante), Spain; Washington State University, United States of America

## Abstract

We theoretically and experimentally analyze the formation of thick Purple Membrane (PM) polyacrylamide (PA) films by means of optical spectroscopy by considering the absorption of bacteriorhodopsin and scattering. We have applied semiclassical quantum mechanical techniques for the calculation of absorption spectra by taking into account the Fano effects on the ground state of bacteriorhodopsin. A model of the formation of PM-polyacrylamide films has been proposed based on the growth of polymeric chains around purple membrane. Experimentally, the temporal evolution of the polymerization process of acrylamide has been studied as function of the pH solution, obtaining a good correspondence to the proposed model. Thus, due to the formation of intermediate bacteriorhodopsin-doped nanogel, by controlling the polymerization process, an alternative methodology for the synthesis of bacteriorhodopsin-doped nanogels can be provided.

## Introduction

Bacteriorhodopsin (bR) is a photochromic protein related to the visual pigment rhodopsin contained in the cone cells of the human retinal, and has been widely explored for its use in electronics and photonic applications [1], [2]. Among several biological molecules, bR has received most attention because of its outstanding optical properties and excellent stability against chemical, thermal and photochemical degradation [1], [2]. In this sense, the use of bR has been proposed and demonstrated for a variety of technological applications in optics such as data storage [1], [3], [4], real-time holography [5], [6], optical display and spatial light modulation [7], [8], optical image processing [9], slow light [10].

bR is the simplest natural light energy transducer and the major protein component of the purple membrane (PM) of the archea *Halobacterium salinarium* [11], [12]. After the absorption of a photon from the visible range (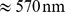) a cyclic sequence of reactions is produced in bR leading to the proton moving from the cytoplasmic side to the intracellular surface and the generation of an electrochemical potential that is used by the archea to maintain its metabolism by driving the synthesis of adenine triphosphate (ATP) [11]. This light-driven photocycle is well-known [13] and the chromophore passes through a sequence of transient optical states, being the sequence of these processes is characterized spectroscopically, defining photocycle intermediates (K, L, M, N, O) that differ in their absorption spectra in the UV-vis (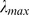 values of 410, 560, and 630 nm for the M, N, and O intermediates respectively) [14], [15].

Absorption spectra of bR in suspensions of purple membrane and in polymeric films have been widely studied as a function of different variables such as pH, temperature, environment, etc [16]–[18]. Asymmetrical Gaussian or Lorentzian bands are found, and analyzed by different phenomenological mathematical expressions [19]–[22]. Furthermore, due to the availability of high resolution crystal structure a better understanding of the properties of these systems has been reached since the spectral and optical properties of these kinds of biomolecules can be determined by the chemical nature of the chromophore, the electronic interactions between the different chromophores, and the interactions between chromophores and their environment [23]–[25].

In 1961, Fano proposed a theoretical treatment of the interaction of a discrete state coupled to a degenerate continuum under the condition that both the discrete and the continuum levels are excited by some external perturbation. As a result asymmetric resonant line shape (Fano profile) associated with absorption by the coupled system is obtained which has been widely used for describing phenomenons throughout nuclear, atomic and solid-state physics, photonic devices, nanoestructures, metamaterials as well as molecular spectroscopy [26], [27].

The aim of this paper is to theoretically and experimentally analyze the formation of PM-polyacrylamide (PM-PA) films from the real-time variation of UV-visible spectra at different pHs. To do so, a model of the formation of those films is proposed and demonstrated by fitting the UV-Visible spectra. In order to explain the behaviour of the UV-Visible spectra of PM-doped polymeric suspension, in our model we have included the scattering of purple membrane and the absorption of bacteriorhodopsin taking into account the previous mentioned Fano profiles. The main reason for this study is to provide a tool for the development of bio-sensitized nanofilms engineered from biomembrane components and inorganic nanoparticles that is a promising field of colloid and interface science and technologies [28]. Recent nano-bioengineering approaches employing quantum dots (QDs) permit the enhancement of the purple membrane (PM) “light-harvesting capacity” compared to native PMs. In this sense, it has been reported several advances for the feasibility of bacteriorhodopsin as biophotosensitizer in excitonic solar cells and nanoscale devices [29], [30]. Requirements of bR-containing nanofilms and nanoparticles are determined by the absorption spectra and, for this, our study is important because explain the resulting anomalies with the medium. Furthermore, Optogenetics is a technology that allows targeted, fast control of precisely defined events in biological systems as complex as freely moving mammals [31]. By delivering optical control at the speed (millisecond-scale) and with the precision (cell type-specific) required for biological processing, optogenetic approaches have opened new landscapes for the study of biology, both in health and disease. This technique has revolutionized the ability to remotely control neurons and also can be used in muscle, and cardiac and embryonic stem cells [32]. For this purpose, in order to deliver light of sufficient intensity to deep structures, absorption and scattering must be characterized with high precision and the presented technique could be also applied in this field [33].

## Theoretical Background

**Figure 1:**
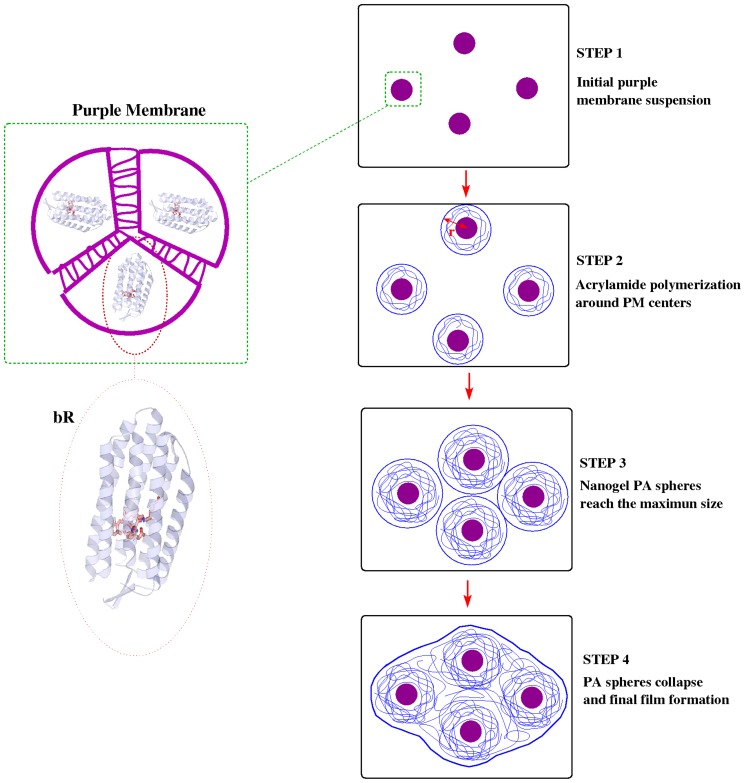
Scheme of the model of formation of PM-polyacrylamide films.

In this paper, we will describe the mechanism of formation of PM-PA film formation from the corresponding PM and acrylamide solutions. We propose the gelification process to be made in four steps as summarized in Figure 1. Basically, when the polymerization initiator system is added to the PM suspension (step 1 in Figure 1) highly reactive radicals are generated which initiate the polymerization reaction of acrylamide. The formation of polyacrylamide chains is located around PM center in a similar manner as the nucleation step in solid state crystal formation (step 2 in Figure 1). As the polyacrylamide chains grow those PM centers increase their size reaching a maximal value, which can be described as PM-PA nanogels (step 3 in Figure 1). Finally, due to the number of PA-centers and their size, these PA spheres collapse to obtain the PM-polyacrylamide film (step 4 in Figure 1).

In order to analyze the process of formation PM-PA film formation we will perform an analysis of the UV-Visible spectra. Thus, the starting point for this study is the equation of the radiative energy transfer given by:
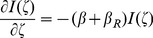
(1)


Where 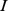 is the intensity, 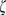 is the propagation coordinate of the electromagnetic field, while 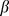 and 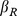 are the macroscopic magnitudes related to the microscopic absorption and scattering cross sections respectively. By integrating Equation 1 with respect to 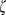, assuming that 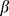 and 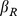 do not depend on 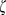 and 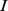 and 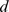 being the thickness of the film, the Optical Density (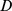) is given by:
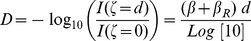
(2)


As it can be deduced from equation 2, the measured optical density depends on two macroscopic quantities, 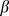 related to bR absorption and 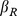 to the PM-scattering. For clarity, we will continue by describing both contributions (absorption and scattering) separately.

### Absorption

The macroscopic parameter 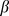 can be obtained from the corresponding microscopic properties by means of Statistical Mechanics according to:
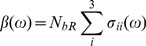
(3)


Where 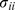 corresponds to the components of the microscopic absorption cross section of the ground state of bR and 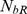 is the population density of bacteriorhodopsin units in the ground state. Due to the complexity of biomolecular systems such as bacteriorhodopsin, it is very difficult to describe the experimental optical spectra. However, quantum mechanical methods are becoming more important for these analyses. Biomolecular spectra is highly complex and, can be understood on the basis of the spectroscopic properties of building blocks, the simplest case being a single chromophore unit which dominates the spectral signature. We propose that, the microscopic absorption cross section of the ground state of bR can be described by two terms (equation 4), the first one corresponding to the chromophore (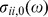) and the second one to the Fano line shape (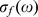) produced by the interaction of a discrete state with a background of continuum of states under the condition that both, the discrete and the continuum levels, are excited by some external perturbation [34]–[36].
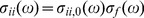
(4)


Thus, when the applied radiation field excite the discrete state and the broad-band system as well, something analogous to the Fano effect [26], [34], can be expected, where the characteristic asymmetric line shape is characterized by the Fano factor 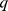. The Fano factor is the ratio of the transition probabilities of the indirect transition and the direct transition into the ground state being the Fano cross section is given by:
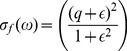
(5)where 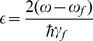, 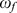 is the Fano resonance frequency and 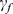 the line width. Equation 5 can be interpreted as interference between the transition into the continuum and the discrete state. It has been satisfactorily employed for justifying the complete optical spectrum of graphene by the excitonic resonance that forms near the van Hove singularity at the saddle point of the band structure and couples to the Dirac continuum [37]; spectral line shapes in both plasmonic and all-dielectric symmetric oligomers [38]; in bulk materials or heavily doped semiconductors in terms of electron-phonon interaction [39]; in nanostructures, where the discrete phonons can interfere with continuum of electronic states available in the material as a result of quantum confinement [40]; for coupled molecules that interact with electron-hole pairs or optical phonons in the substrate [35]. In the case of biomolecules such as bR, the Fano formula has been used in several ways such as the interaction between the chromophore (polyene chain) and protein interaction with the surrounding protein environment or the interaction between chromophores [24], [41]. In this sense, the origin of electronic energy transfer in several photosynthetic pigment-protein complexes involves quantum coherence, which is a property that is directly connected to the intimate details of the medium surrounding the chromophores, due to the decoherence caused by the coupling of the electronic transitions to the fluctuations of the environment [25], [42]. Moreover, experimentally observed and unusually high wavenumber in the IR spectra of the amide I band of bR has been explained by interhelical coupling within a bR monomer [43]. Finally, it is important to add that the visible CD spectrum of bR in purple membrane has a negative CD band at 600 nm and a positive band at 530 nm and has been interpreted by exciton coupling within the bR trimer [44], [45], which is an effect that can also justify the Fano profile.

The microscopic absorption cross section of the ground state is given by [46]:
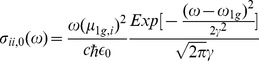
(6)where 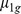 is the ground to excited state transition dipole moment of bR, 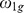 (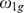 = 2
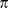
c/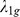) is the frequency of this transition, 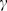 denotes the width of the absorption curve, 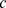 is the light speed and 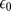 is the dielectric permittivity of the vacuum. In order to assign reliable numerical values to these microscopic parameters we follow two different strategies. On the one hand, we use experimental values present in the literature for 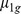 and 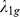 [47], [48], which allow us to have a complete description of the absorption cross section of the system 6. To be exact, the values of the microscopic optical properties of bR were 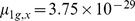 (Cm) and 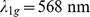 [49].

**Figure 2:**
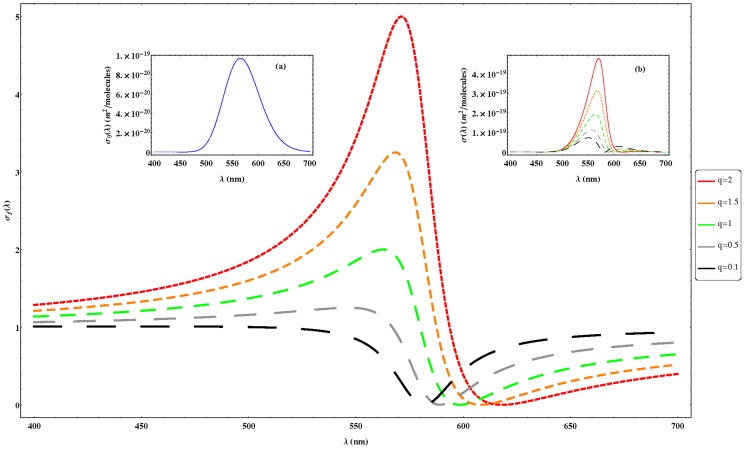
Simulation of 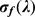 (equation 5) for different Fano parameters (*q*). The inset (a) of the figure represents the microscopic absorption cross section of the ground state (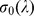) (Equation 6) and the inset (b) the resulting absorption cross section (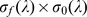) (Equation 4). For these simulations we used the following parameters: 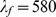 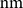, 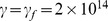 (s^−1^) and 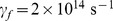, 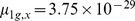 (Cm) and 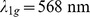.

By introducing equation 5 and 6 in equation 4, we can study the effect on absorption of the two terms shown on the absorption cross section given by equation 4. Figure 2 shows the effect of Fano parameter (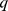) on 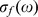, also on 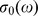 and the resulting 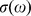 obtained from our model. As can be seen, 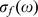 presents the typical Fano profile [34], [36], the line shape being asymmetric with a dip into the background. As 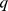 rises, the line shape becomes symmetric Lorentzian (the external perturbation does not couple to the background state) and when 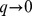 the external perturbation does not couple to the discrete state. The inset (a) of Figure 2 shows the typical microscopic absorption cross section of the ground state (Gaussian line shape) which is slightly asymmetrical at long wavelengths due to the 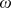 factor (equation 6). Finally, inset (b) describes the resulting absorption cross section (equation 4) as a function of 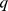 parameter. As can be seen, as 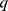 rises the asymmetry to short wavelengths increases as well as a bathochromic shift of the maximum.

### Scattering

Regarding scattering, 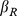, we have assumed that the scatter-particles are spherical with a small radii compared to the wavelength of the scattered light. Thus, 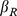 is expressed as a function of the Rayleigh cross section according to [50]:
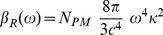
(7)



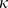
 is a parameter that includes the effect of time-dependent magnitudes during the formation process of the film and it is given by:
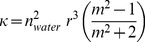
(8)where *r* is the scatter-particle radius, 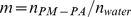 is the ratio of the refractive index of the particle (PM-PA) to that of the surrounding medium (water) and 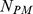 is the population density of PM. As can be deduced from equation 7 as the scatter-particle increases the value of 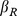 rises, this effect being more significant at short wavelengths. Thus, from the analysis of 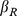, it is possible to obtain information related to the microscopical structure changes produced during the formation of PM-PA films, i.e. the size variation of the scatter-particle during the film formation or the collapse of those particles. In this sense, taking into account equation 8 and the model described in Figure 1, during the formation of PM-PA films the temporal variation of the scattering coefficient is expected to have two different zones. Initially, there is an increase in the values of the scattering coefficient associated to the size of the scatter-particle (*r* variable), which grows due to the polymerization process around the PM centers. As a result, a maximum value of the scattering coefficient is obtained when the scatters reach their maximum size, which can be estimated according to:
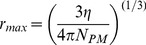
(9)where 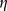 is the maximum packing fraction of hard spheres randomly distributed (around 0.62 [51]) and 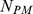 is the density of spheres, which can be assumed to be the same as the number of PM centers. Once the spheres reach the maximum-allowed size, the scattering coefficient begins to decrease due to the collapse of spheres, which produces the approach of the *m*-parameter to the unity and the end of the gelification process. As a result an homogeneous PA medium of PA is obtained instead of water.

Therefore, by using the expressions that describe 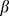 and 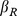 (equations 3–7), the optical density of the system is given by:
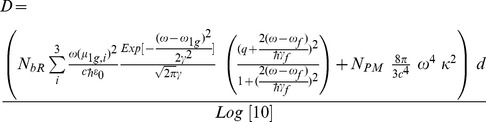
(10)


## Materials and Methods

Purple membrane from Halobacterium salinarum culture were purified following protocol optimized by Oesterhelt and Stoeckenius with minor modifications [52]. Before films processing, PM purity was analyzed by an electrophoresis under denaturing conditions (SDS-PAGE) and absorption spectrum was also measured. We considered the samples with a ratio 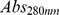/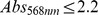 as high-quality. PM-doped films preparation was carried out by using lyophilized PM which were suspended in a solution (10 mg/ml) containing acrylamide-*N,N′*-methylene-bisacrylamide (20%) and Tris(hydroxymethyl)-aminomethane-HCl buffer 0.1 mM at increasing pH (6.0, 7.0, 8.0, 9.0 and 10.0). Each PM-mixture was homogenized using a sonicator (Sonopuls HD 2200) and ammonium persulfate 0.05% (w/v) and *N,N,N′,N′*-tetramethylethyldiamine (1 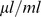) were added for catalyst and initiation of the polymerization reaction. In all films, gel solution was poured in a 1 mm thick cuvette, where the polymerization process occurred. Finally, the temporal evolution of the polymeric film was carried out by using UV-Visible absorption spectrum (400–700 nm) measured by a spectrophotometer (Agilent Tecnologie) every minute for one hour.

## Results and Discussion

**Figure 3:**
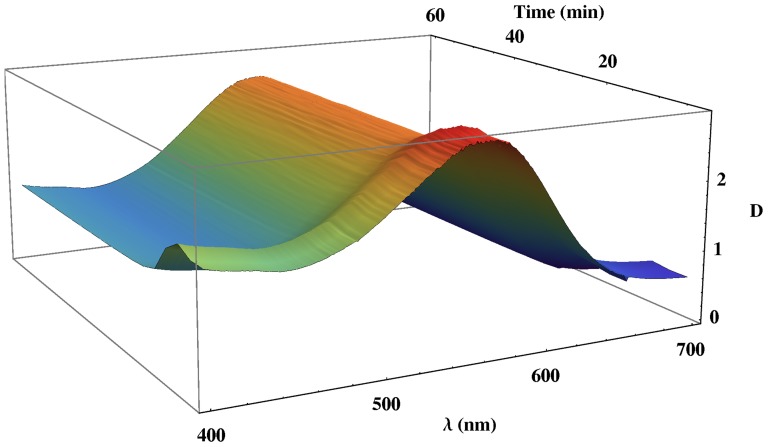
Temporal variation of UV-Visible spectra of the formation of PM-PA films at pH = 7.

**Figure 4:**
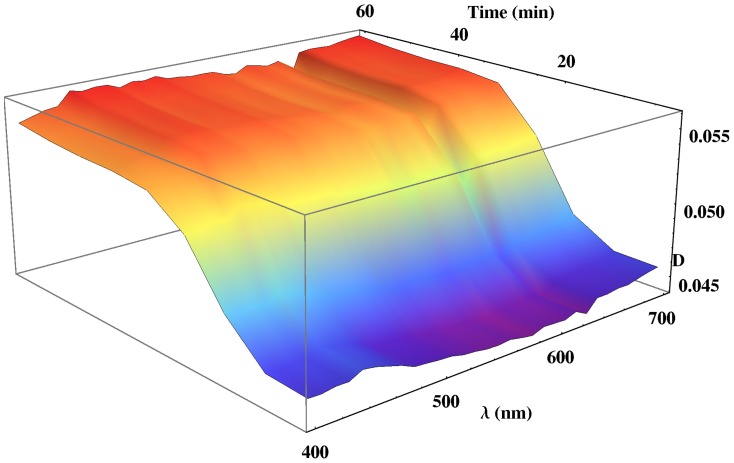
Temporal variation of UV-Visible spectra of the formation of PA films at pH = 7.

In the previous section, we described the mechanism of the formation of PM-PA films from the corresponding PM and acrylamide solutions (Figure 1). In order to prove it, we have performed a real-time analysis of the UV-Visible spectra of the formation of PM-PA film formation at different pHs. As an example, the temporal variation of the UV-Visible spectra at pH = 7 is shown in Figure 3. As can be observed, at the beginning of the process the optical density increases at all wavelengths, this amount being most important at short wavelengths. Furthermore, this effect is observed at all analyzed pHs and depends on PM as demonstrated in Figure 4, where it can be seen that the Optical Density (*D*) in all the spectra region is in absence of PM is seen to be much lower than when PM is present in the material. Thus, the optical density of UV-Visible spectra at pH = 7 without PM is very low and does not present increase previously described in the initial period.

Taking into account these experimental results, we are going to demonstrate the proposed mechanism of the formation of PM-PA films, by using Equation 10. As stated, the theoretical expression of the optical density, has two terms, the effect of bR absorption and scattering due to the PM. Moreover, bR absorption is described by two terms (equation 4), the one corresponding to the chromophore and the Fano line shape given by the interaction of a discrete state with a background of continuum of states. Thus, in order to characterize the absorption and scattering of PM-PA films, in this paper we are going to theoretically analyze the temporal variation of the UV-Visible spectrum (Figure 3) at the pH range of 6.0 to 10.0 by a non-linear fit procedure using equation 10. To do so, 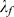 (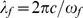), 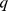, 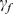, 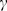 and 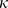 are used as free parameters. Furthermore, regarding to the concentration shown in equation 10, it has been assumed that 75% of the PM weight is bR [53] and the concentrations of bR and PM (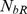 and 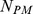) have also been taken as free parameters as 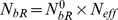 and 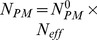 where (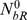 and 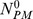) correspond to the initial concentration (experimental) and 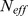 is a factor that takes into account the active concentration (diminution of the initial one) due to possible irreversible changes that occur during the preparation by thermal decomposition, bleaching, polymerization, pH, etc [17].

**Figure 5:**
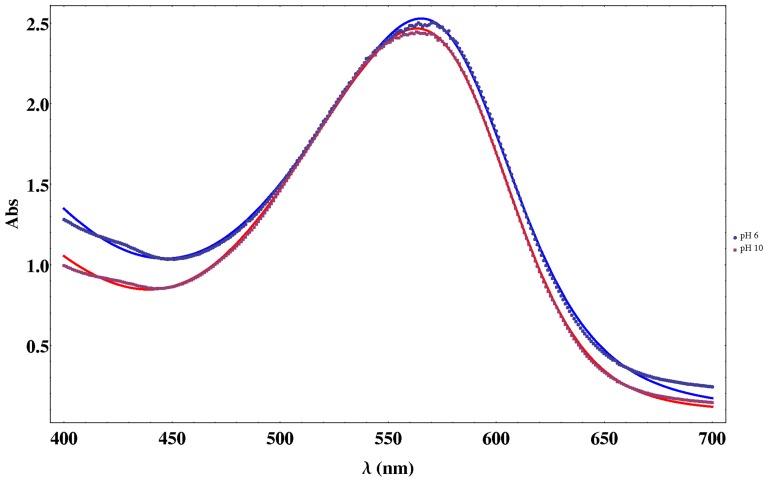
Theoretical (lines) and experimental (points) spectra of PM solutions at pH 6 and pH 10 for a fixed time of 40 minutes.

**Figure 6:**
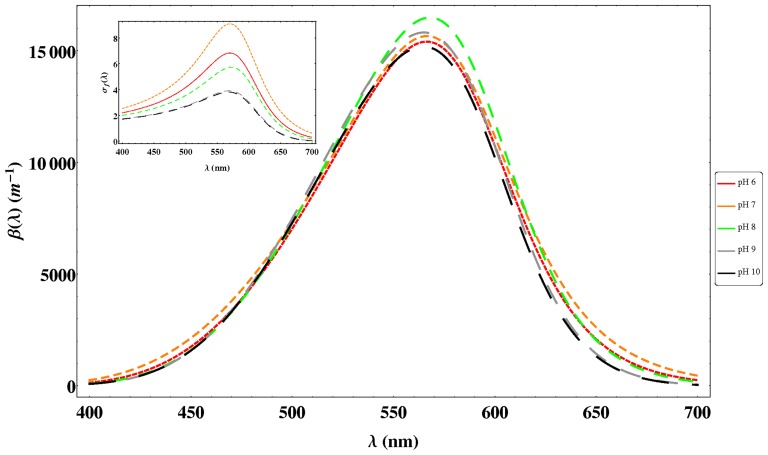
Variation of 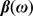 (equation 3) taking into account the parameters obtained as described in Table 1. The inset of the figure represents the contribution of the Fano effect (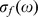) (Equation 5).

In Figure 5, two examples of the experimental and fitted data at pH 6 and 10 are shown for a fixed time of 40 minutes, where the good concordance between theory and experiment (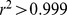) can be seen. In order to measure the effect of pH, by this methodology, the temporal range of between 10 to 60 minutes has been analyzed, and no significant variations are observed in the spectra (Figure 3). Thus, more than 50 experiments at each pH have been fitted, observing that all the free parameters are nearly constant for a fixed pH, the corresponding values obtained at steady-state are shown at table 1. As can be seen the Fano resonance wavelength (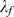) varies between 592 to 602 *nm*, the lower value reached being at pH = 7.0. Regarding to the Fano parameter (*q*), which characterizes the line shape and the asymmetric response, increase from 1.7 to 2.8, where the lowest is given at pH 10 and the highest at pH 7. However, in relation to the Fano effect, the bandwidth is quite similar for all cases, ranging between 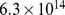 to 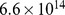. With respect to the bandwidth of the chromophore absorption, this parameter is similar for all cases except for pH = 7, since, in order to show the effect of all these parameters, the corresponding variation of the term associated to the absorption (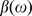) (equation 3) is analyzed for different pH in Figure 6. As it can be seen all the curves are slightly asymmetric and the effect of pH is not important. At pH = 7 a broader absorption response is obtained whereas at higher pH it is sharper. Regarding the maximum value of 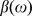 it is observed that a small shift (1 nm) to shorter wavelengths is obtained for pH 6 and 7, 2 nm for pH 9 and 10, meanwhile for pH 8 the shift is 1 nm to longer wavelengths and the highest value. As previously pointed out, 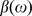 considers the effect of the chromophore and the Fano line shape (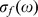) (equation 5) is produced by the interaction of a discrete state with a background of continuum of states. In order to analyze the Fano effect, the inset of Figure 6 shows the variation of 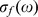 obtained by using the parameters given in table 1. At pH 7 a larger and broader asymmetric shape is obtained which could be produced by the protonation effects of several residues or conformation changes of proteinic structure. Regarding the shift of the maxima, a red shift is observed in all cases with respect to the reference value of 568 nm, the larger one (5 nm) being at pH = 8. Finally, the parameter related to scattering (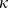) at steady-state is similar at pH 6 and 7 and increases at higher pH values.

**Table 1:** Values of the parameters 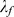, 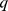, 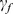 and 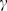 obtained from the non-linear fitting of PM solutions at different pH.

pH	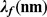	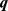	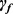 ( 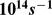 )	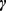 ( 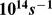 )	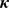 ( 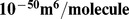 )
6.0	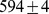	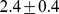	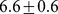	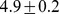	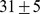
7.0	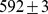	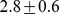	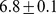	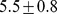	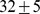
8.0	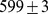	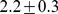	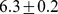	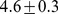	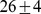
9.0	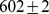	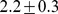	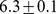	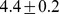	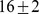
10.0	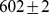	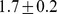	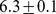	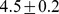	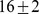

The values correspond to the mean at the stationary state and the error to the standard deviation. 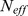 varies between 0.3 to 0.5 for the different cases studied.

**Figure 7:**
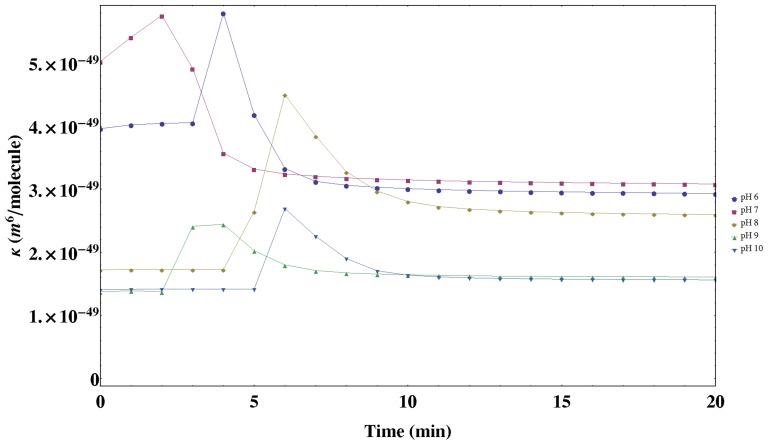
Temporal variation of the 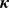-parameter during the polymerization of acrylamide as a function of the pH of the PM solution.

In order to analyze the effects observed in the initial period of polymerization (Figure 3) (non steady-state case) and validate the proposed model of film formation, we are going to perform a complete temporal study of the UV-Visible spectrum. Following a similar methodology to the one given above, by a non-linear fitting procedure using equation 10 the temporal UV-Visible spectra has been analyzed by taking only a time variable parameter (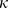) related to the scattering losses of the purple membrane and by using the parameters described in table 1 for each pH (except the 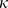 value at the steady-state). As a result, in Figure 7, the temporal variation of the 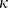 values obtained from the non-linear fit (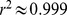) as a function of the pH is analyzed. As can be seen, a similar temporal response is obtained for different pH-solutions; this term associated to scattering is nearly constant at the beginning of the process (a few minutes) and after that a quick amount of scattering is produced until finally a weak decrease is produced reaching a saturation value. The differences among the pH values studied are given by the different value of the maximum scattering reached and the rate of reaching it. Moreover, at pH≤7 the saturation value reached is lower than the initial one, and at pH =  7 scattering increases at the beginning (there is no constant period).

These results can be justified by the proposed model of PM-PA formation (Figure 1). According to Figure 7 the amount of scattering (size of scatter-center) is given by the growth of the polyacrylamide chains around the PM centers similar to the nucleation step in solid state crystal formation. All the scatter-centers increase their size and reorient in order to homogeneously distribute in a minimal energetic configuration. When the size of these PM-doped nuclei are sufficient important, a transition from scatter-center to a network is produced, therefore the scattered-center reaches a maximum size. Thus, as the spatial extension of polyacrylamide increases the scattering term diminishes to a saturation value. Finally, the different temporal behaviour obtained as a function of pH is related to the polymerization rate and the length of the formed polymeric chains which depends on the pH [54]. Note at this point, that taking into account these results, by controlling the polymerization process it is possible to obtain bR-doped nano-gels which could be useful for biomedical and technological applications.

**Figure 8:**
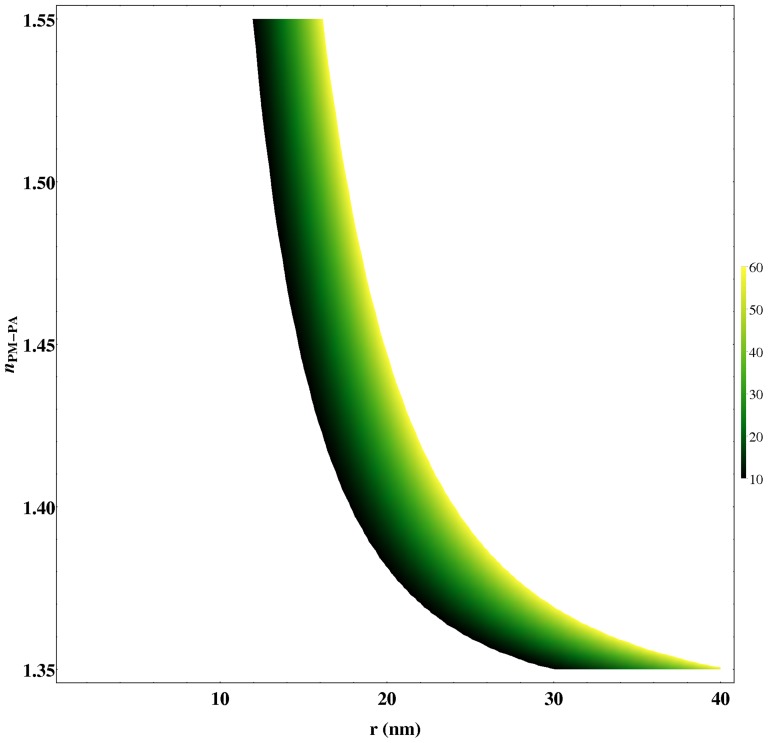
Variation of 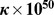 (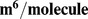) as a function of the PM-PA refractive index and radius obtained by using equation 8 with 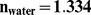 [62].

Finally, according to equation 8, the range of radius (*r*) and the refractive index of PM-PA compatibles to the experimental value of 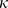 obtained from the fittings are shown in Figure 8. Due to the polymerization process, it is difficult to ensure the refractive index of PM-PA, which could oscillate between (1.35 and 1.55) [55], [56], this figure, therefore, presents an estimation of the radius of PM-PA. As can be seen, a wide range of particle refractive index can be given between 15 to 22 nm. It is important to note that these values are in accordance with the radius sizes estimated by equation 9 for 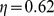 and the used PM concentration used, resulting in scatter particle sizes around 17 to 20 nm. Finally, on the other hand, a larger radius only can be obtained when the refractive index of PM-PA approximates to the water.

## Conclusions

In this study, a model of the formation of thick Purple Membrane-polyacrylamide films has been proposed. During polymerization process of polyacrylamide, polymeric chains grow around purple membrane forming an intermediate PM-doped nanogel. By means of UV-Visible spectroscopy the temporal evolution of the polymerization process of acrylamide for different pH solutions has been experimentally studied. Thus, in order to validate this model, a theoretical treatment has been developed based on semiclassical quantum mechanical techniques for the calculation of absorption spectra. Furthermore, this theoretical analysis takes into account the scattering of the PM and the absorption of bR ground state. However, to describe the observed asymmetrical absorption spectra, Fano line shape has been applied observing a good correspondence between theory and experiment. As a result, the scattering of the PM center and an estimation of its radius has been obtained, verifying that the polymeric chains grow around purple membrane forming an intermediate PM-center. This theoretical analysis of UV-Visible spectra and the possible synthesis route of bR-doped nanogels could have potential applications in biomedicine and photonic technologies. In this sense, challenges exits for preparation of advanced nanogels with novel responsive mechanisms to interact with biological microenvironments. Due to the biocompatibility of the polymers used, the ability to encapsulate and protect purple membrane make these systems attractive for delivery and to use it for example as biosensors [57], [58]. Finally, bacteriorhodopsin-based materials have a broad potential application in photonics [59]. Therefore, we believe that the presented insights, particularly the possible synthesis route of bR-doped nanogels and the theoretical analysis brings the possibility to fabricate nanodevices that could give solution to applications such as organic solar cells, nano optical-switches or optical memories [60], [61].
